# Distribution and metabolism of [^14^C]-resveratrol in human prostate tissue after oral administration of a “dietary-achievable” or “pharmacological” dose: what are the implications for anticancer activity?

**DOI:** 10.1093/ajcn/nqaa414

**Published:** 2021-03-01

**Authors:** Hong Cai, Edwina N Scott, Robert G Britton, Emma Parrott, Ted J Ognibene, Michael Malfatti, Masood Khan, William P Steward, Karen Brown

**Affiliations:** Leicester Cancer Research Centre, University of Leicester, Leicester, United Kingdom; Leicester Cancer Research Centre, University of Leicester, Leicester, United Kingdom; Leicester Cancer Research Centre, University of Leicester, Leicester, United Kingdom; Leicester Cancer Research Centre, University of Leicester, Leicester, United Kingdom; Lawrence Livermore National Laboratory, Livermore, CA, USA; Lawrence Livermore National Laboratory, Livermore, CA, USA; University Hospitals of Leicester NHS Trust, Leicester, United Kingdom; Leicester Cancer Research Centre, University of Leicester, Leicester, United Kingdom; Leicester Cancer Research Centre, University of Leicester, Leicester, United Kingdom

**Keywords:** resveratrol, cancer prevention, tissue distribution, metabolism, pharmacokinetics, prostate, accelerator mass spectrometry, window trial

## Abstract

**Background:**

The dietary polyphenol resveratrol prevents various malignancies in preclinical models, including prostate cancer. Despite attempts to translate findings to humans, gaps remain in understanding pharmacokinetic-pharmacodynamic relations and how tissue concentrations affect efficacy. Such information is necessary for dose selection and is particularly important given the low bioavailability of resveratrol.

**Objectives:**

This study aimed to determine concentrations of resveratrol in prostate tissue of men after a dietary-achievable (5 mg) or pharmacological (1 g) dose. We then examined whether clinically relevant concentrations of resveratrol/its metabolites had direct anticancer activity in prostate cell lines.

**Methods:**

A window trial was performed in which patients were allocated to 5 mg or 1 g resveratrol daily, or no intervention, before prostate biopsy. Patients (10/group) ingested resveratrol capsules for 7–14 d before biopsy, with the last dose [^14^C]-labeled, allowing detection of resveratrol species in prostate tissue using accelerator MS. Cellular uptake and antiproliferative properties of resveratrol/metabolites were assessed in cancer and nonmalignant cell cultures using HPLC with UV detection and cell counting, respectively.

**Results:**

[^14^C]-Resveratrol species were detectable in prostate tissue of all patients analyzed, with mean ± SD concentrations of 0.08 ± 0.04 compared with 22.1 ± 8.2 pmol/mg tissue for the 5 mg and the 1 g dose, respectively. However, total [^14^C]-resveratrol equivalents in prostate were lower than we previously reported in plasma and colorectum after identical doses. Furthermore, resveratrol was undetectable in prostate tissue; instead, sulfate and glucuronide metabolites dominated. Although resveratrol reduced prostate cell numbers in vitro over 7 d, the concentrations required (≥10 µM) exceeded the plasma maximum concentration. Resveratrol mono-sulfates and glucuronides failed to consistently inhibit cell growth, partly due to poor cellular uptake.

**Conclusions:**

Low tissue concentrations of resveratrol species, coupled with weak antiproliferative activity of its conjugates, suggest daily doses of ≤1 g may not have direct effects on human prostate.

This trial was registered at clinicaltrialsregister.eu as EudraCT 2007-002131-91.

## Introduction

Resveratrol (*trans*-3,4’,5-trihydroxystilbene) is a naturally occurring antifungal found in a variety of foods including the skin of red grapes and peanuts (1). Since the seminal report that resveratrol could inhibit multiple stages of carcinogenesis (2), it has been the subject of extensive preclinical investigations for the treatment and prevention of numerous pathologies including cancer, cardiovascular and Alzheimer's disease, insulin resistance, and diabetes (3). Attempts to translate these findings to humans have increased in recent years (4–6) but there are still fundamental gaps in our understanding of the pharmacokinetic-pharmacodynamic relations and how plasma/target tissue concentrations correlate with clinical efficacy or beneficial effects on health maintenance (7). Such information is needed to inform the choice of optimum dose and formulation requirements, and to enhance understanding of the key modes of action for resveratrol in each pathology (8); it is also particularly important given the notoriously low systemic bioavailability of parent resveratrol due to its rapid metabolism, which, if not taken into consideration, would affect its clinical utility.

Resveratrol has been shown to prevent the development of a variety of malignancies in preclinical rodent models, including colorectal and prostate cancers (9). Concentrations of parent resveratrol achievable in human colon after oral ingestion of 1 g daily can be as much as 100- to 1000-fold higher than in plasma, and appreciable concentrations are also detected after a far lower intake of 5 mg (10, 11). Furthermore, the concentrations generated in human colorectal tissue by these doses have been found to elicit changes in pharmacodynamic markers that may be mechanistically linked to cancer-preventive effects of resveratrol (11, 12). In addition, concentrations of ∼5 nmol/g tissue (equivalent to ∼5 µM, assuming 1 g tissue has a volume of 1 mL) have been reported in colorectal liver metastases from patients who received 5 g SRT501/d, a micronized formulation of resveratrol designed to improve bioavailability (13). This dosage caused a significant increase in cleaved caspase-3, a marker of apoptosis, in malignant tissue. Resveratrol metabolites, but not the parent compound, have been detected in resected breast tissue from women undergoing surgery for breast cancer who took capsules containing a mixture of fruit and cocoa extracts plus resveratrol for ∼6 d (14).

Beyond these 3 cases, concentrations of resveratrol and its metabolites in internal tissues distant to the gastrointestinal tract have not yet been defined in humans, but may be significantly different because they are reliant on systemic delivery with no contribution of topical absorption through direct contact with the contents of the gut lumen. This is important because much of the in vitro evidence demonstrating anticancer effects of resveratrol in prostate models emanates from studies that required concentrations ≥5 µM for activity, and often employed as much as 25–100 µM, which may not be attainable in human prostate tissue (15–18). The relevance of mechanistic insights gained using high resveratrol concentrations may therefore be questionable.

To evaluate the potential clinical utility of resveratrol for the prevention and/or treatment of prostate cancer we conducted a trial to ascertain whether resveratrol reaches detectable concentrations in prostate target tissue and define the metabolite profile. To date, clinical trials of resveratrol have used a wide dose range, from as little as 5 mg, which can be considered potentially achievable through dietary sources, up to 5 g daily. Current toxicity and tolerability data, together with a potential for drug interactions, suggest the maximum daily dose for long-term use should be 1 g (19, 20). In this study, we compared the distribution of 5 mg with that of 1 g resveratrol, after daily administration for ≤2 wk. We then assessed the uptake and antiproliferative effects of resveratrol and metabolite mixtures in a panel of cancer and nonmalignant prostate cell lines, to determine whether clinically achievable concentrations are likely to have direct biological activity in humans.

## Methods

### Chemicals and reagents

All chemicals and solvents were purchased from Sigma-Aldrich and Fisher Scientific unless stated otherwise. Cell culture materials were obtained from Thermo Fisher Scientific Limited. HPLC columns and accessories were supplied by Waters Limited. The resveratrol used in the cell-based studies was a gift from Royalmount Pharma. Standards of resveratrol-3-glucuronide, resveratrol-4’-glucuronide, and a 3:2 mixture of resveratrol-3- and 4’-sulfate were synthesized according to our established protocols and the structures verified by HPLC, MS, and ^1^H-NMR (21). DU145 and LNCaP prostate cancer cell lines and PNT2 immortalized human prostate normal cell line were purchased from the European Collection of Authenticated Cell Cultures.

### Clinical trial

The study was approved by the UK Medicines and Healthcare products Regulatory Agency, the Liverpool UK Research Ethics Committee, the University Hospitals of Leicester National Health Service (NHS) Trust Research and Development Department, the Institutional Review Board at Lawrence Livermore National Laboratory, and the Administration of Radioactive Substances Advisory Committee. The trial was conducted according to the Helsinki II Declaration and Good Clinical Practice and was registered on the European Clinical Trials Database (EudraCT number 2007-002131-91). The trial protocol is available at: https://www2.le.ac.uk/centres/cancer/people/14CResveratroltrialprotocolversion8.pdf.

Resveratrol manufactured to standards of Good Manufacturing Practice was obtained from Orchid Chemicals and Pharmaceuticals Ltd. and supplied through Royalmount Pharma. Hard gelatin capsules containing either 5 mg or 250 mg resveratrol were formulated by Nova Laboratories. [^14^C]-Resveratrol was synthesized by BioDynamics Research Ltd. and radiodiluted with unlabeled resveratrol (Royalmount Pharma). This material was then used by Pharmaceutical Profiles to manufacture [^14^C]-resveratrol capsules, which contained 5 mg total resveratrol and gave a labeled dose of 44.5 kBq (0.962 µSv equivalent dose). For the last dose before biopsy, patients in the 1 g group received 4 × 250 mg resveratrol capsules plus a single 5 mg [^14^C]-resveratrol capsule, affording a total dose of 1.005 g. The additional 5 mg of resveratrol was considered negligible for those taking 1 g daily.

### Trial participants

Patients suitable for the study were identified at multidisciplinary team meetings; they were eligible if they required prostate biopsies for suspected prostate cancer or for monitoring of existing low-grade cancer, or surgery for the management of benign prostatic hypertrophy. Patients were >18 y of age and were asked to avoid food and drink containing resveratrol during the trial. Participants also completed a food diary and dosing calendar to monitor compliance. Exclusion criteria included excessive alcohol intake, chemotherapy or use of an investigational drug within 4 wk of tissue sampling, any malabsorption syndrome, chronic use of warfarin or antiepileptic drugs, and evidence of abnormal renal or liver function. All patients were recruited at the University Hospitals of Leicester NHS Trust, where they were randomly allocated into 3 parallel groups of 10 that received either 5 mg resveratrol daily, 1 g (4 × 250 mg) resveratrol daily, or no intervention (control patients), as **Supplemental Figure 1** shows. There was no formal randomization method employed; patients were chronologically allocated to each of the 3 groups in turn. To maximize the chances of detecting resveratrol species we employed the sensitive technique of accelerator mass spectrometry (AMS) (22). The control group was primarily included to provide tissue for measurement of background concentrations of radiocarbon naturally present in human prostate, which is needed for the AMS analysis (23). Participants were assessed for adverse events and graded in accordance with the National Cancer Institute Common Terminology Criteria for Adverse Events.

### Trial design

The primary objective of this nonblinded phase 1 trial was to compare concentrations of resveratrol and its metabolites in prostate tissue after ingestion of a “pharmacological” and a “dietary” dose. No formal sample size calculation was performed for this study because there were no relevant human data available to inform a power calculation. Ten patients per group were deemed appropriate to investigate the primary objective, because we have previously defined resveratrol pharmacokinetics in plasma and colorectal tissue with 10 participants/dose (10, 24). Two extra prostate biopsies were taken for trial purposes and both were needed for AMS analysis.

Samples were collected on ice and stored at −80°C until analysis. The majority of tissue samples were analyzed whole by AMS, but HPLC-AMS analysis was also performed on tissue extracts from 1 patient/group to determine representative concentrations of resveratrol and its metabolites. Because of the amount of tissue available it was only possible to conduct 1 type of analysis (whole tissue or HPLC-AMS) for each patient. A further 2 randomly selected tissue samples per group were retained in case technical failures during the HPLC-AMS procedure meant an additional sample had to be analyzed (see the participant flow diagram in Supplemental Figure 1).

### AMS analysis

Samples were prepared in a designated laboratory, free from extraneous ^14^C-contamination, at the University of Leicester. Prostate tissue (∼8 mg) samples were then subject to AMS at Lawrence Livermore National Laboratory according to standard protocols (23). Tissue was analyzed neat (without carrier) to measure the concentration of total [^14^C]-resveratrol species; this involved converting samples to elemental carbon in the form of graphite, which was then analyzed up to 7 times for radiocarbon content or until the measurement variation was within ±5%. The amount of ^14^C due to resveratrol/metabolites was calculated by subtracting the mean background concentration of radiocarbon detected in prostate tissue from untreated control patients. The concentration of [^14^C]-resveratrol equivalents was then determined by taking into account the radioisotope specific activity, tissue mass analyzed, and percentage of total carbon in prostate tissue (8.75%) determined by elemental analysis. The term “resveratrol equivalents” covers all [^14^C]-labeled resveratrol species (i.e., the parent compound and metabolites) and refers to the fact that concentrations are calculated using the molecular mass of resveratrol.

### HPLC-AMS analysis

Metabolite profiles were established by HPLC-AMS analysis of methanol and acetone extracts of homogenized tissue, according to our standard methods (10). The method was originally adapted from our validated HPLC-UV assay but is not validated in its present form in conjunction with AMS analysis. The organic extracts were concentrated to dryness, then reconstituted in water:methanol (1:1) and separated by HPLC on a Waters Atlantis C_18_ column (4.6 mm × 150 mm, 3 μm, maintained at 35°C) preceded by a Waters Atlantis guard C_18_ column (4.6 mm × 20 mm, 5 μm). A flow rate of 1 mL/min was employed with a linear gradient and mobile phases of 2% isopropanol in 5 mM ammonium acetate and 2% isopropanol in methanol, as previously described (11). Fractions were collected every minute during each HPLC run and concentrated to dryness, then reconstituted in 50:50 methanol:water (200 μL) before AMS analysis. HPLC fractions were supplemented with a carbon carrier (1 μL tributyrin, equivalent to 615 μg carbon) to provide sufficient carbon mass for sample preparation and AMS analysis. Samples were then analyzed as detailed above; results are presented as Fraction Modern (22, 23). The lowest limit of detection (LOD) for [^14^C]-resveratrol species by HPLC-AMS analysis, calculated as the mean plus twice the SD of background concentrations of radiocarbon in HPLC fractions, was 0.003 and 0.5 pmol resveratrol equivalents/mg tissue in a single fraction, for the 5 mg and the 1 g dose group, respectively. The values are different for each group because the extent of radioisotope dilution (fraction labeling) was different for each dose amount.

### Cell culture

DU145, LNCaP, and PNT2 cell lines were maintained in Roswell Park Memorial Institute 1640 media containing 10% fetal calf serum under standard conditions in 5% CO_2_ at 37°C. To determine the effects of resveratrol/metabolite exposure on proliferation, cells were seeded at densities ranging from 3000 to 8000 cells per well in 24-well plates. Cells were treated on either a single occasion or a daily basis with freshly prepared solutions of resveratrol (final concentrations 0.01–50 µM) and its sulfate or glucuronide metabolites (0.01–250 µM) for a maximum of 7 d and cell numbers were determined using a Coulter counter (ZM model, Beckman Coulter). All experiments were performed in triplicate on 3 separate occasions. Cells were also treated daily for 7 d with a mixture of resveratrol and its major metabolites designed to mimic the concentrations previously reported in human plasma from our clinical trials in which healthy volunteers received 5 mg or 1 g resveratrol (11, 21). These concentrations were 0.1 and 12.5 µM for the 4’-glucuronide, 0.2 and 15.6 µM for the 3-glucuronide, 0.21 and 28 µM for the sulfate mixture, and 0.003 and 0.5 µM for resveratrol, at the 5 mg and 1 g doses, respectively.

To measure the intracellular uptake of resveratrol/metabolites, cells were cultured in 175-cm^2^ flasks and cell pellets and media were collected pretreatment and then at intervals of 5, 15, 60 min, and 24 h after applying resveratrol (10 µM) or its metabolites (75 µM). Cells were washed 3 times in PBS, counted, and pellets were stored, along with aliquots of media, at −80°C until analysis. These incubations were conducted in phenol red–free medium and concentrations were determined using our established HPLC-UV assay outlined below (21, 25). Basal amounts of organic anion-transporting polypeptide 1B3 (OATP1B3) protein expression across the cell lines were assessed by western blotting using an anti-OATP1B3 antibody (Abcam #ab139120) and actin as a loading control (Santa Cruz Biotechnologies #sc-1616).

### Intracellular uptake of resveratrol and metabolites

Cell pellets were extracted with acetone (250 µL) and the supernatant was concentrated to dryness under N_2_, then reconstituted in methanol:water (1:1, 100 µL). After centrifuging, 20 µL of the supernatant was injected onto the HPLC column. For measurement of resveratrol and its metabolites, calibration curves were constructed by spiking blank cell pellets with each analyte and extracting using the standard protocol. Measured intracellular concentrations were normalized according to cell numbers and converted to picograms per milligram of cells.

The HPLC conditions for determination of resveratrol and its metabolites in cell pellets were reported previously and were based on an assay validated for the analysis of human tissue (21, 25). Briefly, the HPLC system consisted of a Waters Alliance 2695 separations module with detection wavelength set at 325 nm. HPLC separation was carried out on a Waters Atlantis dC_18_ column (150 × 4.6 mm, 3 μm) at a flow rate of 1 mL/min, with a gradient program using 2% isopropanol in water containing 5 mM ammonium acetate as mobile phase A and 2% isopropanol in methanol as mobile phase B.

### Statistical analysis

Data were assessed for normality using the Kolmogorov–Smirnov test and appropriate parametric or nonparametric tests selected. For the clinical trial, when comparing patient demographics (age) across 3 groups, ANOVA with a Dunnett's post hoc test was used. Statistical significance between the 2 resveratrol groups was determined by a 2-tailed unpaired Student's *t* test for data that were normally distributed, or a Mann–Whitney *U* test for nonparametric data, when comparing days of resveratrol intervention, interval between [^14^C]-resveratrol dose and surgery, and concentrations of [^14^C]-resveratrol in prostate tissue. In addition, the Pearson *r* between duration of resveratrol and prostate [^14^C]-resveratrol concentrations was calculated. For the laboratory experiments examining the effect of resveratrol and its metabolites on cell number relative to a control incubation, significant differences were identified using 1-factor ANOVA with a Dunnett's post hoc test. Data in graphical form are presented as mean values ± SEM or SD as specified in the figure legends. All statistical analysis was performed using Prism version 8 (GraphPad Software, Inc.).

## Results

### Patient demographics and safety of resveratrol

Characteristics of the 30 patients recruited to the trial such as age, ethnicity, performance status, and diagnosis were similar across treatment groups (**Table 1**); there was no significant difference in patient age across the 3 groups, or in the time between [^14^C]-resveratrol dose and sampling for the 2 intervention groups. **Supplemental Table 1** lists concomitant medications. All but 1 individual completed the study; this patient was on low-dose resveratrol and stopped after 2 d owing to migraine, which he was known to suffer from. It was considered unlikely that the headaches were due to resveratrol. Consistent with findings from other trials, resveratrol was generally well tolerated with no serious adverse events reported. The only likely side effects potentially caused by resveratrol were low-grade diarrhea and abdominal pain, which were each experienced by a single patient on the 1 g dose (**Table 2**). The duration of intervention ranged from 7 to 15 d, with the mean being 13 and 11 d for the 5 mg and the 1 g group, respectively. The range arises owing to unavoidable differences in the interval between patients being invited for a biopsy and having the procedure done. There was a significant difference in the duration of resveratrol intervention between the 2 groups (*P* < 0.05, Mann–Whitney *U* test); however, this would not be expected to affect the outcome because all participants took resveratrol for ≥1 wk, which was the minimum target to allow for any effect of resveratrol on drug-metabolizing enzymes, before the final ^14^C-labeled dose. Consistent with this prediction, there was no evidence that duration of intervention with this once-daily dosing regimen significantly influenced tissue concentrations of [^14^C]-resveratrol species, within each group (**Supplemental Table 2**). In total, 19 patients received a [^14^C]-radiolabeled dose of resveratrol, on average ∼3 h before their biopsy.

**TABLE 1:** Patient demographics^1^

	Control (no resveratrol)	5 mg resveratrol	1 g resveratrol
Patients, *n*	10 recruited, 10 completed	10 recruited, 9 completed	10 recruited, 10 completed
Age, y	66.3 ± 8.0 (56–83)	63.4 ± 7.2 (52–78)	60.9 ± 6.0 (51–68)
Ethnicity	9 Caucasian,1 Indian/Caucasian	10 Caucasian	10 Caucasian
PS	PS 0 = 8, PS 1 = 1, PS 2 = 1	PS 0 = 7, PS 1 = 1, PS 2 = 1	PS 0 = 10
Final histological diagnosis	Benign (*n* = 3), PIN (*n* = 1), Gleason 6 (*n* = 2), Gleason 7 (*n* = 2), Gleason 9 (*n* = 1), Gleason 10 (*n* = 1)	Benign (*n* = 1), Gleason 6 (*n* = 5), Gleason 7 (*n* = 2), Gleason 8 (*n* = 1)	Benign (*n* = 2), PIN (*n* = 1), Gleason 6 (*n* = 4), Gleason 7 (*n* = 2), Gleason 9 (*n* = 1)
Time between [^14^C]-resveratrol dose and sampling, h	Not applicable	2.35 ± 0.71 (1.5–3)	2.85 ± 0.54 (2.3–4)
Days treated with resveratrol,* *n*	Not applicable	13.3 ± 3.8 (11–15)	10.9 ± 2.6 (7–14)

^1^Values are mean ± SD (range) unless indicated otherwise. The timing between administration of the last dose and tissue sampling was determined by when patients arrived for their procedure and their order on the theatre list. *Significant difference in the duration of resveratrol intervention between the 2 dose groups (*P* < 0.05, Mann–Whitney *U* test). There was no statistically significant difference between the groups for patient age (ANOVA with a Dunnett's post hoc test) or interval between the [^14^C]-resveratrol dose and tissue sampling (Student's *t* test). PIN, prostate in situ neoplasia; PS, Performance Status.

**TABLE 2:** Adverse events reported, in accordance with the National Cancer Institute CTC for Adverse Events^1^

Adverse event	Patients (dose group, severity as per CTC grading)	Diagnosis	Causality
Diarrhea	*n* = 1 (1 g, CTC grade 1)	Side effect of resveratrol	Possibly caused by resveratrol
Abdominal pain	*n* = 1 (1 g, CTC grade 1)	Side effect of resveratrol	Possibly caused by resveratrol
Cough	*n* = 1 (5 mg, CTC grade 1)	Viral infection	Not caused by resveratrol
Hyperbilirubinemia (at baseline)	*n* = 1 (1 g, CTC grade 1);*n* = 1 (control, CTC grade 1)	Gilbert's syndrome	Not caused by resveratrol
Elevated alanine transaminase (at baseline)	*n* = 1 (5 mg, CTC grade 1)	Exercise	Not caused by resveratrol
Hyperkalemia (at baseline)	*n* = 1 (control, CTC grade 2)	Normal physiological variation	Not caused by resveratrol
Headache	*n* = 1 (5 mg, CTC grade 2)	Migraine	Probably not related

^1^CTC, Common Terminology Criteria.

### [^14^C]-Resveratrol species reached target prostate tissue in patients

**FIGURE 1:**
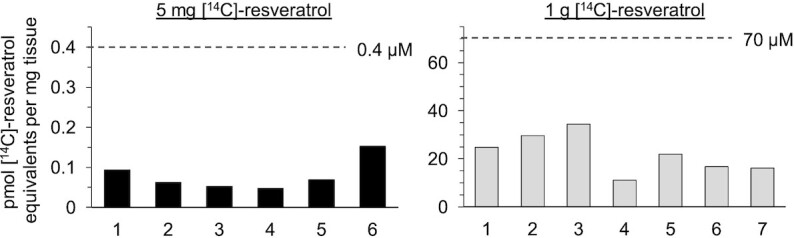
Concentrations of [^14^C]-resveratrol equivalents in human prostate tissue after a low dietary achievable dose or high pharmacological dose. Patients received either 5 mg (*n* = 6) or 1 g (*n* = 7) oral resveratrol daily for up to 2 wk before surgery, with the last dose being [^14^C]-radiolabeled (44.5 kBq, 0.962 µSv). Tissues were obtained a mean of ∼3 h after the [^14^C]-resveratrol dose and the radiocarbon concentration was measured using AMS. There was a significant difference in mean prostate concentrations between the 2 dose groups (*P* < 0.0001, Student's *t* test). For comparison, the dashed lines show the mean plasma concentration of [^14^C]-resveratrol equivalents detected 3 h after ingestion of a single dose (5 mg or 1 g) by healthy volunteers; these values are taken from our previous trial which involved analogous methodology (11).

[^14^C]-Resveratrol species were detectable in all patient tissues analyzed, with a ∼280-fold difference in concentrations (mean ± SD: 0.08 ± 0.04 compared with 22.1 ± 8.2 pmol/mg tissue for the 5 mg and 1 g dose, respectively; *P* < 0.0001, Student's *t* test), reflecting the 200-fold difference in dose (**Figure 1**, Supplemental Table 2). When compared with our previous plasma pharmacokinetic study in healthy volunteers who received the same [^14^C]-labeled oral doses of resveratrol, the prostate concentrations of total [^14^C]-resveratrol equivalents were lower than the mean plasma concentrations at a similar time point (3 h) after capsule ingestion (Figure 1) (11).

**FIGURE 2:**
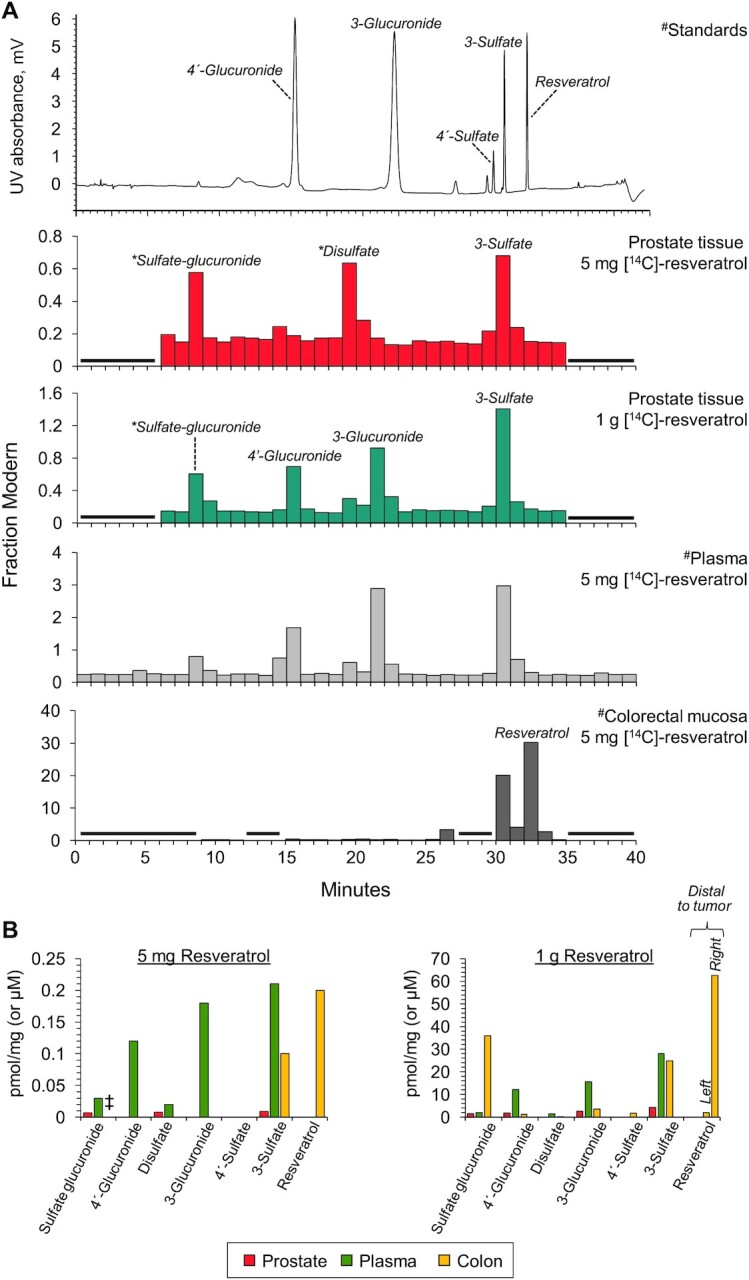
Metabolites of [^14^C]-resveratrol in human prostate tissue detected by HPLC-AMS and comparison with plasma and colorectal profiles. (A) Prostate tissue metabolite profiles determined by HPLC-AMS analysis of selected samples from 1 patient on each resveratrol dose. ^#^Also included are previously published plasma and colorectal tissue profiles from healthy volunteers and colorectal cancer patients, respectively, given 5 mg [^14^C]-resveratrol, and a UV chromatogram from the analysis of authentic metabolite standards (11). These chromatograms are from Cai et al. Sci Transl Med. 7, 298ra117 (2015) and are reprinted with permission from AAAS. Peaks designated by * were assigned on the basis of their chromatographic properties and our previous LC-MS/MS analysis (21), because synthetic standards were not available. (B) Comparison of the achievable concentrations of resveratrol and its metabolites in human prostate, colorectal tissue, and plasma after ingestion of 5 mg or 1 g [^14^C]-resveratrol. For the 1 g dose, large variations in resveratrol concentrations have been observed between tissues arising from the right and left sides of the colon, therefore separate values have been included for tissue taken distal to the tumor. Concentrations are expressed as pmol/mg, which approximates to µM, assuming that 1 g tissue equates to 1 mL. Values for plasma and colorectal tissue (the green and yellow bars, respectively, in B) are from Cai et al. (11) and Patel et al. (21). ^‡^Indicates that the concentration of resveratrol sulfate-glucuronide was not previously analyzed in colorectal tissue of patients that received a 5 mg dose, whereas concentrations have been determined in patients that took 1 g resveratrol. AMS, accelerator mass spectrometry.

To ascertain which resveratrol species were responsible for the radiocarbon detected in prostate tissue, samples from 2 patients, 1 on each dose, were extracted and subjected to an HPLC fractionation step before AMS analysis. The reconstructed chromatograms are shown in **Figure 2**A, along with a UV trace of authentic standards and [^14^C]-chromatograms reproduced from our previously published analysis of a plasma sample and colorectal tissue from individuals that received 5 mg [^14^C]-resveratrol, for direct comparison of the metabolite profile (11). Parent resveratrol was not detected in prostate tissue from either patient (LOD equals 0.003 and 0.5 pmol/mg tissue for the 5 mg and 1 g dose groups, respectively); instead, conjugated metabolites dominated, with prominent peaks due to resveratrol 3-sulfate, disulfate, and sulfate-glucuronide derivatives at the low dose. In addition, resveratrol 3- and 4’-glucuronides were also present in tissue from the patient who took 1 g. This pattern mirrors the plasma profile in humans, in whom oral resveratrol is rapidly metabolized, and contrasts with our previous finding in colorectal samples, where resveratrol itself is a major species and persists in the tissue long after ingestion (Figure 2) (10, 11).

Further comparison across tissues and plasma suggests that relatively low concentrations of resveratrol species reach the prostate (Figure 2B). This may be partly attributed to the difference in time point postdosing for plasma compared with prostate (1 h compared with 3 h) but the discrepancy still holds when comparing total [^14^C]-concentrations detected at similar times (3 h) (Figure 1).

### Activity and uptake of resveratrol metabolites in prostate cells

**FIGURE 3:**
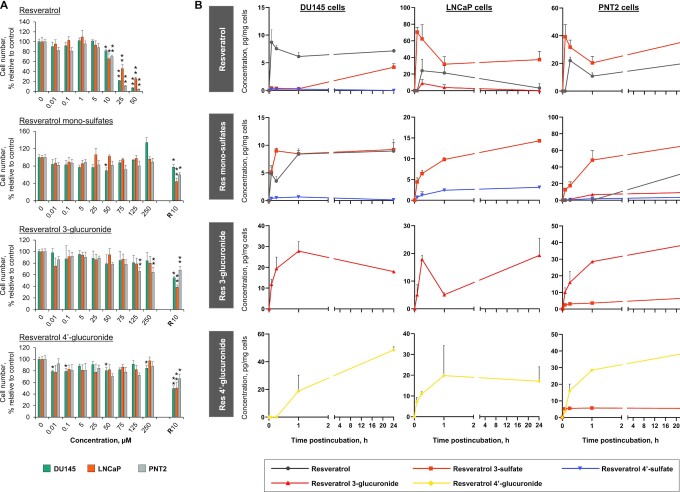
Antiproliferative effects and cellular uptake of resveratrol and its metabolites across a panel of prostate cell lines. (A) Proportion of cells remaining, relative to solvent control, after incubation with resveratrol or its metabolites (a mono-sulfate mixture, 3-*O*-glucuronide, or 4’-*O*-glucuronide) for 7 d (mean + SEM of 3 experiments, performed in triplicate). ^*,**^Significant difference with the control incubations: *P < 0.05, **P < 0.005 (1-factor ANOVA). In the metabolite incubations, resveratrol (10 µM) was included as a positive control (indicated by R10). (B) Change in intracellular resveratrol-related species in 3 prostate cell lines over 24 h, after addition of resveratrol (10 µM), resveratrol mono-sulfates (75 µM), resveratrol-3-*O*-glucuronide, or resveratrol-4’-*O*-glucuronide (both at 75 µM). Concentrations were determined by HPLC-UV analysis and data represent the mean + SD of 3 independent experiments.

To investigate the potential for resveratrol species to directly interfere with the development and proliferation of cancer cells in prostate tissue we assessed the ability of resveratrol and its sulfate and glucuronide conjugates to reduce cell numbers, measured 7 d after application of a single treatment, across a panel of prostate cancer (DU145, LNCaP) and normal epithelial (PNT2) cell lines (**Figure 3**A). The aim was to compare, for each individual metabolite at each concentration, the effect relative to the solvent control in the 3 cell lines. A wide concentration range was used to encompass concentrations measured in prostate tissue in this study and previously reported in human plasma (21, 24). Higher concentrations that could conceivably be achieved using alternative formulations of resveratrol which improve absorption and/or extend the half-life were also included. To aid interpretation of differential effects we measured cellular uptake over 24 h (Figure 3B, **Supplemental Figure 2**).

At concentrations ≥10 µM a single addition of parent resveratrol to culture media caused a significant reduction in cell numbers across all cell lines after 7 d, with the proportion remaining decreasing with dose. The IC_50_ values for each cell type were relatively consistent, ranging from 22 to 30 µM (**Supplemental Figure 3**), and resveratrol was equally potent in normal and malignant cell lines. In comparison, the resveratrol mono-sulfate mixture had minimal effects on cell number, even though the concentrations tested were much higher (≤250 µM) to reflect the higher in vivo plasma concentrations of resveratrol conjugates. There was no consistent inhibitory effect in any of the cell lines relative to the solvent control. Resveratrol 4’-glucuronide also lacked evidence of convincing activity over the wide concentration range examined. Resveratrol 3-glucuronide reduced the number of PNT2 cells by ∼35% at concentrations of 125 and 250 µM (*P* < 0.005), but there were no significant effects in the 2 cancer cell lines.

Analysis of the intracellular uptake and metabolite profiles over 24 h provided potential explanations for the differential activity (Figure 3, Supplemental Figure 2). Parent resveratrol was rapidly taken up by all 3 cell lines and generated peak concentrations in the range ∼10–20 pg/mg cells. Universally, it was efficiently metabolized to resveratrol 3-sulfate, with quantitatively lower concentrations of resveratrol-4’-sulfate and resveratrol-3-glucuronide detectable only in DU145 and LNCaP cells, respectively. The concentration of resveratrol used in this uptake study was 10 µM, which had a significant, albeit relatively small, effect on cell numbers across all lines, causing an 18%–34% reduction by 7 d. Using this approach the possibility cannot be ruled out that some of the resveratrol species detected were adhering to the cell surface rather than within the cells, although efforts were made to remove external material through washing. Incubations with the conjugates were performed at a higher, but still clinically achievable, concentration (75 µM), to account for higher systemic concentrations of resveratrol metabolites in humans. All 4 metabolites were taken up by the 3 cell lines, suggesting the presence of active transport mechanisms across the different cell types. Notably, concentrations detected within cells were quantitatively low given the higher exposures than for resveratrol itself (75 compared with 10 µM). Incubation with resveratrol-sulfates led to the rapid generation of resveratrol in DU145 cells, whereas it was only detectable at 24 h in PNT2 cells; in contrast, no parent was formed in LNCaP cells, implying a lack of sulfatase enzymatic activity. Although single resveratrol mono-glucuronides were detected intracellularly in their respective incubations, there was no conversion to resveratrol, and there was also an apparent lack of glucuronidase activity in DU145 and LNCaP cells, which resulted in an absence of any newly formed metabolites. In contrast, resveratrol 3-sulfate was generated as the only metabolite in PNT2 cells incubated with either glucuronide, which suggests active glucuronidase and sulfotransferase enzymes in this normal cell line.

### Repeated exposures and effect of clinically achievable mixtures

**FIGURE 4:**
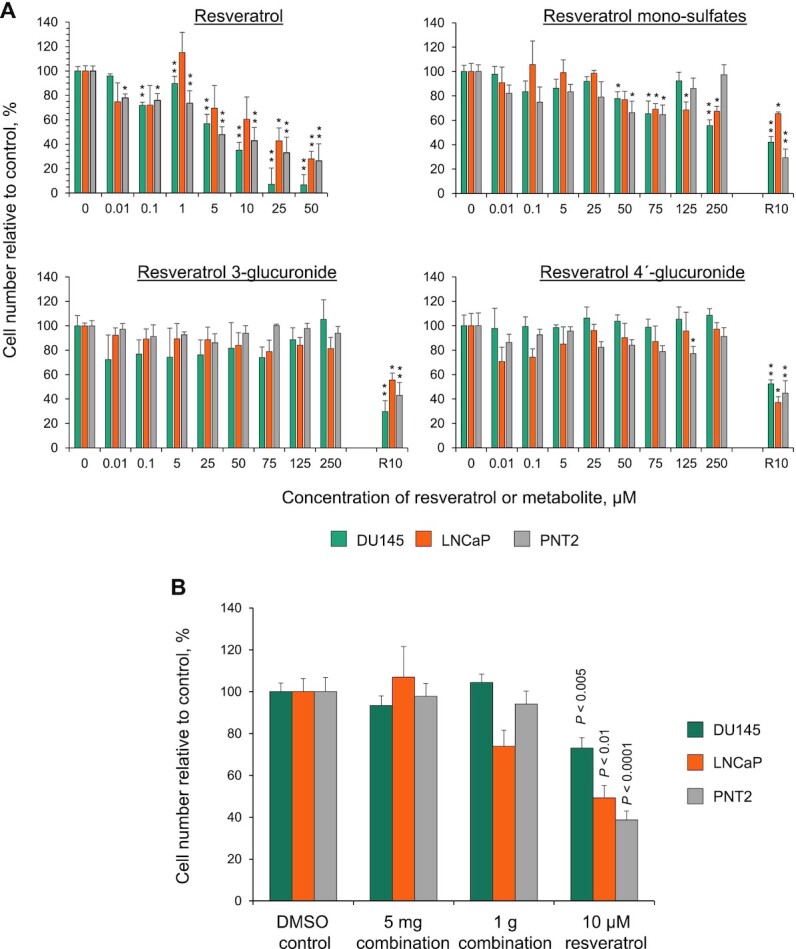
Effect of repeated exposure and a clinically achievable combination of resveratrol and its metabolites on cell proliferation. (A) Proportion of cells remaining, relative to solvent control, after repeated daily exposure to resveratrol or its metabolites (a mono-sulfate mixture, 3-*O*-glucuronide, or 4’-*O*-glucuronide) for 3 d (mean + SEM of 3 experiments, performed in triplicate). ^*,**^Significant difference with the control incubations: *P < 0.05, **P < 0.005 (1-factor ANOVA with Dunnett's post hoc test). In the metabolite incubations, resveratrol (10 µM, R10) was included as a positive control. (B) Cells were also treated daily for 7 d with a mixture of resveratrol and its major metabolites designed to mimic the concentrations previously reported in human plasma from our clinical trials in which healthy volunteers received 5 mg and 1 g resveratrol (11, 21, 24). These concentrations were 0.1 and 12.5 µM for 4’-glucuronide, 0.2 and 15.6 µM for 3-glucuronide, 0.21 and 28 µM for the sulfate mixture, and 0.003 and 0.5 µM for resveratrol, at the 5 mg and 1 g doses, respectively. Significant differences compared with the control incubations were identified using ANOVA with a Dunnett's post hoc test.

To explore the effects of repeated exposure, cells were treated daily with fresh resveratrol or metabolites for 72 h (**Figure 4**). This regimen failed to increase the activity of glucuronide metabolites, but parent resveratrol caused significant reductions in cell numbers from the lower concentration of 0.01 µM and the sulfate mixture had slightly enhanced potency, with significant effects from 50 µM, although there was no real increase in activity over the range 50–250 µM. To investigate the potential for additive effects of combinations, cells were also cultured for 7 d in the presence of a mixture containing resveratrol plus metabolites that was reflective of the plasma concentrations produced after ingestion of the low (5 mg) and the high (1 g) dose of resveratrol by patients in our previous clinical trials (11, 21, 24) (Figure 4B). Despite adding in the mixture fresh each day, the 5 mg concentration had no effect on cell number, and although the 1 g mixture caused a 26% reduction in LNCaP cells this difference was not statistically significant; the other 2 cell lines remained unaffected.

We have previously shown that the degree of uptake of resveratrol sulfate metabolites by colorectal cancer and normal cells correlates with the relative expression of OATP1B3 membrane transporters, although other unidentified transporters are also likely to contribute (21). The role of OATP1B3-mediated transport in the uptake of resveratrol 3-sulfate and resveratrol disulfate has also been reported by Riha et al. (26). Comparison of OATP1B3 basal protein expression across the untreated prostate cell lines revealed no associations with conjugate uptake; in fact, DU145 cells, which contained the lowest intracellular concentrations of resveratrol 3-sulfate, displayed the highest expression of this transporter, perhaps suggesting that other transporters may be more important in prostate cells (**Supplemental Figure 4**).

## Discussion

A significant body of preclinical evidence suggests resveratrol may have value in the treatment and/or prevention of prostate cancer (18, 27–29). Our clinical trial has shown that although resveratrol species can reach prostate tissue after both a dietary-achievable and a pharmacological dose, the metabolites rather than parent resveratrol predominate, suggesting this tissue mirrors the plasma kinetic profile, albeit with lower total concentrations detected (range: 11–34 µM and 0.05–0.15 µM for 1 g and 5 mg, respectively) than systemic concentrations at the same time point postdosing (Figures 1, 2, Supplemental Table 2). This contrasts with our previous analysis of colorectal mucosa where the concentrations of total [^14^C]-resveratrol equivalents reached the order of 100–600 pmol/mg in certain patients after ingestion of a 1 g dose (11). Hence, tissue bioavailability of total resveratrol species appears to be ∼10-fold higher in the colon than in the prostate, which is consistent with regions of the gastrointestinal tract containing the highest concentrations of resveratrol derivatives after oral administration to rodents (30). The absence of detectable parent resveratrol in prostate tissue in our study is in agreement with the profile detected in breast tissue of patients who took capsules containing a complex mixture of fruit and cocoa extracts plus resveratrol before surgery (14). A daily dose of 162 mg resveratrol generated quantifiable concentrations of sulfate and glucuronide metabolites but free resveratrol was not observed. The authors suggested that this may be due to the analytical Ultra Performance Liquid Chromatography-MS-based assay having lower sensitivity for resveratrol, but it could equally be because the unconjugated compound does not reach or persist in breast tissue (14). Our results support the latter theory for human prostate, because AMS analysis measures the ^14^C:^13^C ratio and has the same LOD for all [^14^C]-labeled resveratrol species (23).

In vitro studies describing the anticancer activity of resveratrol in prostate cells have typically required concentrations of ≥5 µM to elicit inhibitory effects on proliferation and the underlying processes (15–17). Although there are accounts of proapoptotic activity at lower concentrations of 2 µM (29), this is still probably beyond the concentrations of parent resveratrol likely to occur in human prostate at a dose of 1 g, assuming tissue availability is a reflection of circulating plasma concentrations (24). Furthermore, our findings suggest any free resveratrol is likely to be short-lived in prostate tissue, and essentially undetectable, or below the LOD of 0.5 pmol/mg for the 1-g dose, by ∼3 h. These results therefore raise the question of whether the very low concentrations of resveratrol generated in plasma after oral dosing can have activity in prostate tissue or whether the major metabolites are taken up into cells and have intrinsic activity, or are able to regenerate resveratrol intracellularly as has previously been described in colorectal cancer cells for sulfate conjugates.

A concentration of ≥10 µM resveratrol was needed to cause a significant reduction in cell numbers across our panel of prostate cell lines after a single application, and this led to peak cellular concentrations of ∼10–20 pg/mg, which are similar to the concentrations previously detected in colorectal cancer cells treated under the same protocol (21). However, when the colorectal cancer cell lines were incubated with resveratrol (10 µM), much higher cellular concentrations of resveratrol sulfate (330–360 pg/mg) were generated; this difference between parent and metabolite was not seen in the prostate cells and suggests considerably lower amounts of resveratrol species may be entering the cells. The lack of a consistent dose-dependent reduction in prostate cell number associated with resveratrol glucuronide treatment for 7 d, and also after repeated application for 3 d, may be explained by inefficient uptake of these metabolites and a lack of detectable intracellular conversion to parent resveratrol. This reinforces the inactivity of glucuronide metabolites, at least with respect to direct antiproliferative activity in cellular systems (21).

Similarly, there was a lack of any convincing growth-inhibitory effect with resveratrol mono-sulfates, which contrasts with our previous study in which these metabolites caused a significant concentration-dependent reduction in colorectal cancer cell number (21). The difference may be attributed to far greater uptake of these conjugates by the colorectal cancer cells, leading to intracellular generation of resveratrol in HT-29 cells at concentrations ≥5-fold higher than those detected in the prostate cell panel.

The low tissue concentrations of resveratrol species detected in human prostate coupled with the weak antiproliferative activity of its conjugates suggest resveratrol at doses of ≤1 g may not have direct anticancer effects on prostate cells in vivo. This theory is upheld by the only randomized controlled trial of resveratrol published to date with immediate relevance to prostate cancer. It involved middle-aged men with metabolic syndrome, and although the primary objective was to examine the effects of resveratrol on bone, the investigators also measured prostate size, prostate-specific antigen (PSA), and hormonal markers to explore its potential role for the management of benign prostate hyperplasia (BPH) and prostate cancer (31, 32). After a 4-mo intervention at a dose of 150 mg or 1 g daily, resveratrol had no effect on prostate size, PSA, or concentrations of testosterone, free testosterone, or dihydrotestosterone in serum. Consequently, the authors concluded there was no evidence to recommend resveratrol for the treatment of BPH. However, resveratrol was found to reduce the concentration of androgen precursors in a dose-dependent manner, with the higher 1 g intake decreasing androstenedione and significantly reducing dehydroepiandrosterone (DHEA), as well as halving the concentration of DHEA sulfate relative to the control group. Androgens, which are produced by the testes with a minor contribution from the adrenal glands, play an important indirect role in the pathogenesis of both BPH and prostate cancer growth. In vitro cell studies suggest resveratrol inhibits the 17,20 lyase activity of Cytochrome P450 17A1, which leads to a concentration-dependent decrease in DHEA secretion (33). However, it has been speculated that the resveratrol concentrations attained in human testes after systemic administration may be insufficient to block this enzyme (31, 34). Our findings from the present pharmacokinetic study are consistent with this hypothesis and indicate that a greater understanding of the systemic effects of resveratrol on metabolic processes that may indirectly influence prostate carcinogenesis is needed.

We further propose that for tissues that rely on the systemic circulation for drug delivery, such as the prostate and breast (14), where resveratrol metabolites predominate, the use of formulations designed to improve bioavailability may be necessary for efficacy. This contrasts with cancers of the gastrointestinal tract where orally administered resveratrol can be directly absorbed from the lumen (9, 11). Various delivery systems have been developed to increase resveratrol bioavailability including nanoencapsulation in lipid nanocarriers or liposomes, nanoemulsions, micronization, insertion into polymeric particles, and nanocrystals (35). These types of formulations, when given at a relatively high dose, may offer a way of achieving concentrations in the target tissue associated with direct antiproliferative and proapoptotic activity in preclinical cellular models. However, most of these products have not yet been tested in humans, so trials to evaluate safety and define the pharmacokinetic-pharmacodynamic relation in target tissue would be needed before larger studies could be justified for prostate cancer.

In conclusion, the low bioavailability of intact resveratrol may limit its clinical utility in the prevention and treatment of prostate cancer unless higher concentrations can be achieved in the target tissue without compromising its excellent safety profile. The possibility also remains that systemic effects of resveratrol in other organs may indirectly modulate the growth and proliferation of malignant prostate cells and further investigation of potential mechanisms is warranted in clinically relevant models that take into account the pharmacokinetic considerations highlighted in this study.

## Supplementary Material

nqaa414_Supplemental_File

## Data Availability

Data described in the article will be made available upon written request, subject to application.

## Notes

Supported by Cancer Research UK programme grant C325/A13101 (to KB) and Leicester Experimental Cancer Medicine Centre grants C325/A15575 and C10604/A25151 (to WPS). Accelerator MS analysis was performed at the National Resource for Biomedical Accelerator Mass Spectrometry, which is operated at Lawrence Livermore National Laboratory under the auspices of the US Department of Energy under contract DE-AC52-07NA27344. The Resource is supported by the NIH, National Institute of General Medical Sciences, and Biomedical Technology Research Resources under grant P41GM103483 (to TJO). Unlabeled resveratrol was a gift from Royalmount Pharma. None of the funders had any role in any aspect of the study.

Supplemental Tables 1 and 2 and Supplemental Figures 1–4 are available from the “Supplementary data” link in the online posting of the article and from the same link in the online table of contents at https://academic.oup.com/ajcn/.

HC and ENS contributed equally to this work.

Abbreviations used: AMS, accelerator mass spectrometry; BPH, benign prostate hyperplasia; DHEA, dehydroepiandrosterone; LOD, limit of detection; NHS, National Health Service; PSA, prostate-specific antigen; OATP1B3, organic anion-transporting polypeptide 1B3.
