# Supplementary material for: Distribution and metabolism of [14C]-resveratrol in human prostate tissue after oral administration of a “dietary-achievable” or “pharmacological” dose: what are the implications for anticancer activity?
Source: Am J Clin Nutr. 2021 Mar 1;113(5):1115–25. doi: 10.1093/ajcn/nqaa414 (PMC8106746; doi:10.1093/ajcn/nqaa414)
Supplement: nqaa414_Supplemental_File [file nqaa414_supplemental_file.docx]

**On-line supporting material**

**Distribution and metabolism of [^14^C]-resveratrol in human prostate tissue after oral administration of a ‘dietary-achievable’ or ‘pharmacological’ dose: what are the implications for anticancer activity?**

Hong Cai, Edwina N Scott, Robert G Britton, Emma Parrott, Ted J. Ognibene, Michael Malfatti, Masood Khan, William P. Steward, Karen Brown.

**Supplementary Table 1. Concomitant medications taken by the patients in each group**

|  | **Number of patients** | | |
| --- | --- | --- | --- |
| **Drug** | **Control** | **5 mg Resveratrol** | **1 g Resveratrol** |
| Adcal | 2 |  |  |
| Amlodipine | 1 | 2 | 1 |
| Aspirin | 2 | 5 | 1 |
| Atenolol |  |  | 1 |
| Atorvastatin |  | 1 |  |
| Bendrofluazide |  |  | 1 |
| Bimatoprost |  | 1 |  |
| Bisoprolol | 1 |  |  |
| Candesartan |  |  | 1 |
| Digoxin | 1 |  |  |
| Ezetimibe | 1 |  |  |
| Finasteride |  | 2 |  |
| Fludrocortisone |  |  |  |
| Folic Acid |  | 1 |  |
| Gaviscon | 1 |  |  |
| Glicazide | 1 |  | 1 |
| Lacidipine |  |  | 1 |
| Lansoprazole | 2 | 2 |  |
| Lisinopril |  | 2 | 1 |
| Metformin | 2 |  | 1 |
| Paracetamol | 3 | 2 | 2 |
| Methotrexate |  | 1 |  |
| Omeprazole |  |  | 1 |
| Paroxetine |  | 1 |  |
| Pergabalin |  |  | 1 |
| Prednisolone |  | 1 |  |
| Quinine |  | 1 |  |
| Ramipril |  | 1 | 1 |
| Ranitidine | 1 |  |  |
| Risedronate | 1 |  |  |
| Rizatripan |  |  | 1 |
| Seretide inhaler |  | 3 | 1 |
| Simvastatin | 3 | 3 | 2 |
| Sumatriptan |  | 1 |  |
| Tamsulosin |  | 5 | 3 |
| Telmisartan |  | 1 |  |
| Tramadol/codeine | 3 | 2 | 2 |
| Ventolin inhaler |  | 3 | 1 |
| Zoplicone |  | 1 |  |
|  |  |  |  |

|  |  |  |  |
| --- | --- | --- | --- |

**Supplementary Table 2. Tissue concentrations of total [^14^C]-resveratrol species in patients that received 5 mg or 1 g resveratrol daily.**

| **Patient ID** | **Total [^14^C]-Resveratrol equivalents**  **(pmols/mg tissue)*** | **Days of Resveratrol or [^14^C]-Resveratrol ingestion^a^** | **Time between**  **[^14^C]-Resveratrol dose**  **& surgery (hours)** | **Pathological tumour stage** |
| --- | --- | --- | --- | --- |
| **5 mg Resveratrol** | | | | |
| **P030** | 0.094 | 14 | 2.75 | Gleason 7 |
| **P035** | 0.062 | 14 | 2 | Gleason 6 |
| **P054** | 0.052 | 11 | 2.33 | Benign |
| **P067** | 0.048 | 13 | 3 | Gleason 8 |
| **P029** | 0.069 | 14 | 3 | Gleason 6 |
| **P031** | 0.153 | 14 | 3 | Gleason 6 |
| **1.0 g Resveratrol** | | | | |
| **P038** | 27.77 | 10 | 2.67 | Gleason 7 |
| **P049** | 29.72 | 14 | 4 | Gleason 9 |
| **P051** | 34.35 | 7 | 3 | Gleason 7 |
| **P052** | 11.07 | 8 | 2 | Gleason 6 |
| **P055** | 21.89 | 11 | 2.5 | Benign |
| **P061** | 16.67 | 12 | 2.67 | Prostate *in situ* neoplasia |
| **P063** | 16.17 | 10 | 2.33 | Benign |

*pmol/mg tissue is equivalent to µM concentrations, calculated for ease of comparison with plasma and *in vitro* concentrations, by assuming that 1 g tissue is equivalent to 1 mL.

Patient IDs listed above correspond to numbers 1-6 and 1-7 in the bar charts of Figure 1, for the 5 mg and 1 g dose groups, respectively.

^a^There was no significant correlation between the number of days of resveratrol ingestion and concentrations of [^14^C]-resveratrol measured in prostate tissue; the Pearson correlation coefficient (r) was 0.033 (p=0.94) and 0.48 (p=0.34) for the 1 g and 5 mg dose groups, respectively.

**
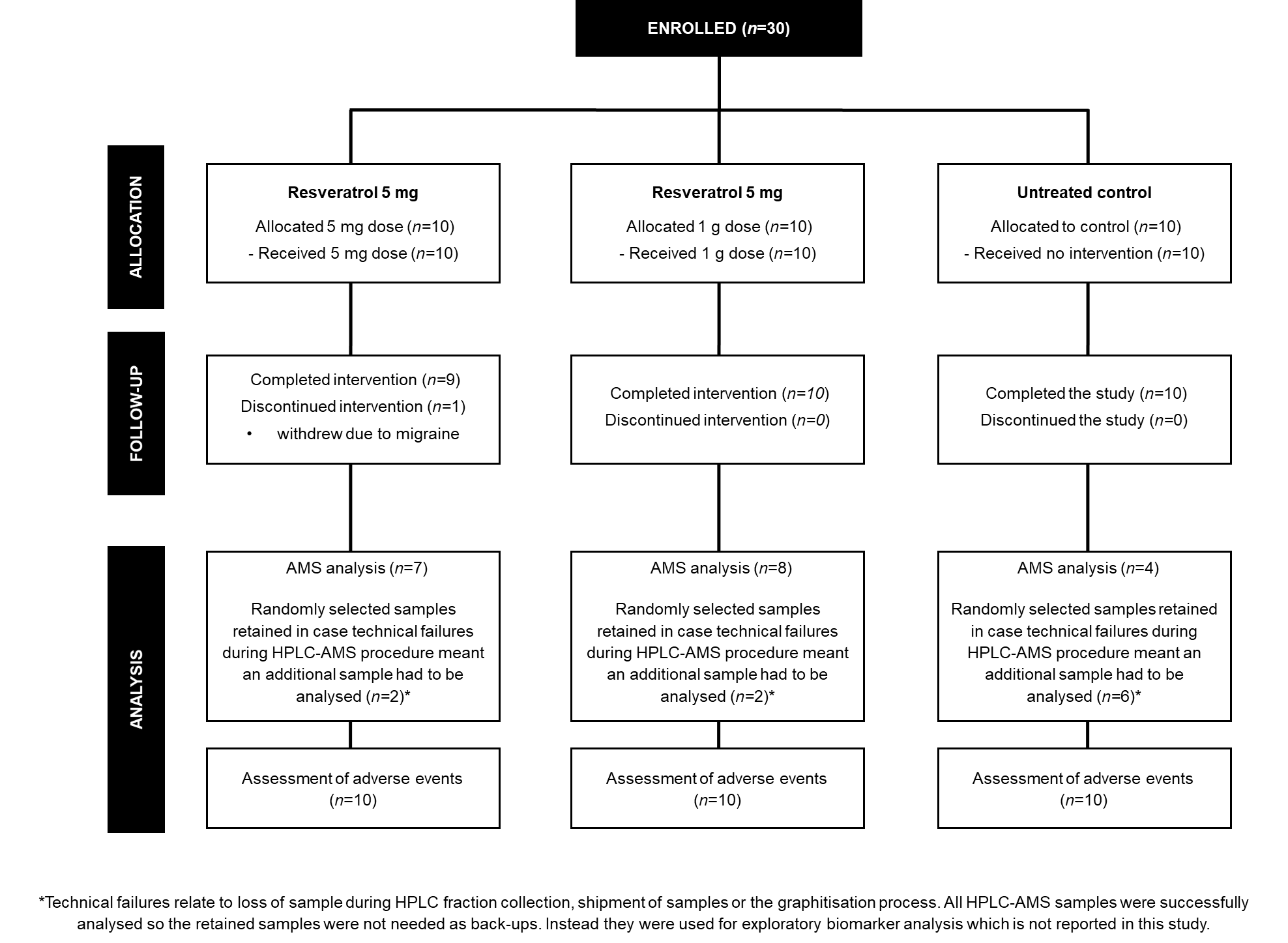
**

**Supplementary Figure 1.** Participant flow diagram for the clinical trial.


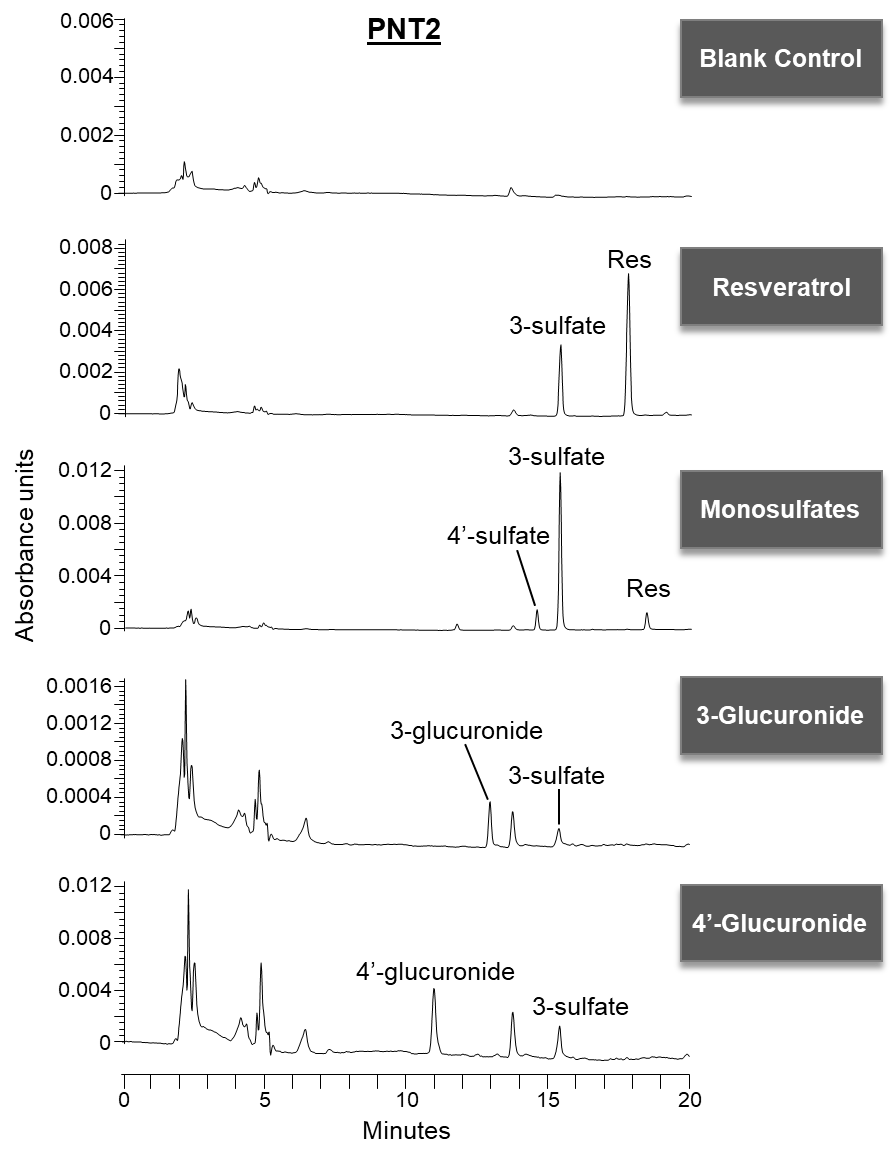


**Supplementary Figure 2.** Representative HPLC-UV chromatograms illustrating the profile of intracellular resveratrol species detected in PNT2 cells 24 h after addition of resveratrol or its conjugated metabolites to the incubation (as indicated in the boxes). The detection wavelength was 325nm.

**
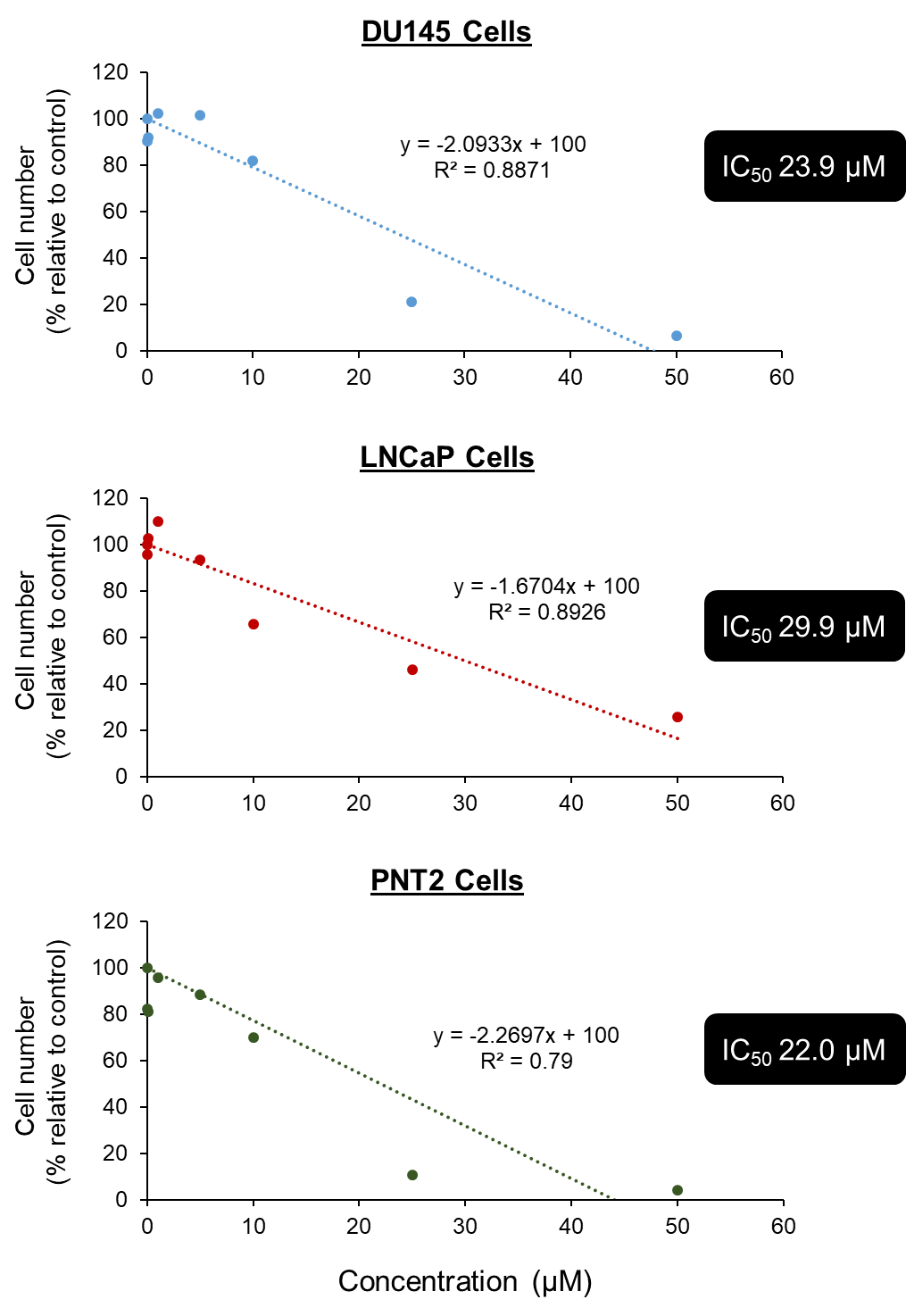
**

**Supplementary Figure 3. Determination of resveratrol IC_50_ concentrations across the three prostate cell lines.** Cells were exposed to resveratrol or solvent vehicle only over a concentration range of 0.01-50 µM for 7 days and were then counted.

**Supplementary Figure 4. Comparative baseline expression of the membrane transporter OATP1B3 in the prostate cancer and normal cell lines.** HT-29 colorectal cancer cells were included as a positive control.
